# Pesticide exposure and lung cancer risk: A case-control study in Nakhon Sawan, Thailand

**DOI:** 10.12688/f1000research.24114.1

**Published:** 2020-06-02

**Authors:** Teera Kangkhetkron, Chudchawal Juntarawijit

**Affiliations:** 1 Department of Natural Resources and Environment, Faculty of Agriculture, Natural Resources and Environment, Naresuan University, Phitsanulok, 65000, Thailand; 2 Nakhon Sawan Provincial Public Health Office, Minstry of Public Health, Muang District, Nakhon Sawan, 60000, Thailand; 3 Department of Natural Resources and Environment, Faculty of Agriculture, Natural Resources and Environment, Naresuan University, Muang District, Phitsanulok, 65000, Thailand

**Keywords:** Lung cancer, Pesticides exposure, Herbicides, Insecticides, Fungicides

## Abstract

**Background:** Pesticide exposure might increase risk of lung cancer. The purpose of this study was to investigate the association between the historical use of pesticides commonly found in Thailand, and lung cancer.

**Methods:** This case-control study compared a lifetime pesticide exposure of 233 lung cancer cases, and 458 healthy neighbours matched for gender, and age (±5 years). Data on demographic, pesticide exposure, and other related factors were collected using a face-to-face interview questionnaire. Associations between lung cancer and types of pesticides as well as individual pesticides were analysed using logistic regression adjusted for gender (male, female), age (≤54, 55–64, 65–74, ≥75), cigarette smoking (ever, never smoke), occupation (farmer, non-farmer), and exposure to air pollution (yes, no).

**Results:** It was found that lung cancer was positively associated with lifetime use of herbicides, insecticides, and fungicides. Compared to people in the lowest quartile of number of days using the herbicides and insecticides, those in a higher quartile had an elevated risk of lung cancer, with odds ratio (OR) between 2.79 (95% confidence interval (CI) 1.12–6.95), and 28.43 (95% CI 11.11-72.76) (p < 0.001). For fungicides, only the most exposed group had a significant risk (OR = 4.97; 95% CI 1.49-16.56). For individual pesticides, those presenting a significant association with lung cancer were dieldrin (OR = 2.76; 95% CI 1.42-5.36), chlorpyrifos (OR = 3.98; 95 % CI 2.06-7.67), and carbofuran (OR = 2.58; 95% CI 1.48-4.51).

**Conclusions:** The results showed that lung cancer among Thai people in Nakhon Sawan province is associated with previous pesticide use. This finding was consistent with previous studies in other parts of the world. Further study should focus on identifying more individual compounds that may cause lung cancer, as well as other types of cancer.

## Introduction

Lung cancer is a common and deadly type of cancer. In 2018, there were 2.1 million people around the world diagnosed with lung cancer, and 1.8 million died of the disease ^1^. In 2018, Thailand had 170,495 incidences, and 114,199 deaths of lung cancer ^2^. Besides genetic factors ^3^, a major risk factor of lung cancer is cigarette smoking ^4, 5^. However, lung cancer was also related to other risk factors, including asbestos, crystalline silica, radon, polyaromatic hydrocarbons, diesel engine exhaust particles, chromium, and nickel ^6, 7^. Recent studies have also linked cooking fumes to lung cancer ^8^.

Pesticide exposure might also cause lung cancer ^9^. The association between pesticides and lung cancer were presented around 50 years ago among grape farmers ^10^. A large study in the United States found that lung cancer cases increase with the number of years working as a licensed pesticide applicator ^11^. Another study in USA reported an increased risk of lung cancer among acetochlor herbicide users (RR = 1.74, 95% CI 1.07-2.84) ^12^. In Pakistan, a study also found a strong association between pesticide exposure and lung cancer (OR = 5.1, 95% CI 3.1-8.3) ^13^.

Some studies can also link individual pesticides to lung cancer. In the USA, a study evaluated 50 pesticides and found that seven—dicamba, metolaclor, pendimethalin, carbofuran, chlorpyrifos, diazinon, and dieldrin—to be positively associated with lung cancer ^14^. Another study also showed a significantly increased risk of lung cancer among applicators who had been exposed to dieldrin ^15^. Jones *et al.* ^16^ reported an increased lung cancer incidence among male pesticide applicators with the highest exposure category of diazinon (odds ratio (OR) = 1.6, 95% confidence interval (CI) 1.11-2.31). Other individual pesticides that had been associated with lung cancer were chlopyrifos ^17^, diazinon ^18^, pendimenthalin ^19^ and carbofuran ^20^ **.**

To our knowledge, the association between lung cancer and pesticides has never been studied before among Thai people. The objective of this study was to investigate associations between pesticide exposure and lung cancer among people living in Nakhon Sawan province, Thailand. The results can be used for the prevention of lung cancer, and to support the global literature.

## Methods

### Study participants

This study is a population-based case-controlled study. Cases referred to people diagnosed with lung cancer during the period of January 1, 2014 to March 31, 2017, and having residence in Nakhon Sawan province, Thailand. Cases were selected from the database of Cancer Based Program (TCB) operated by Thai National Cancer Institute ^21^. From 299 living cases registered during the study period, 229 cases (77%) were contacted in person, and participated in this study. Controls were neighbours who did not have lung cancer, but were of the same gender, and age (±5 years) as the cases. In each case, two controls were randomly selected by the interviewer. In this study, data from 458 controls were used as a comparison group.

The minimum sample size was determined to be 229 for cases and 558 for controls using Kelsey’s formula ^22^ (unmatched population base case-control study). The assumptions used were as follows: proportion of case with pesticide exposure was 0.5 ^23^, proportion of control with exposure was 0.4 ^24^, and the ratio of case to control was 1:2 ^25^.

### Questionnaire

Data on pesticide exposure and other risk factors were collected using a questionnaire previously used in a study on pesticide exposure and diabetes ^26^. The questionnaire has two major parts (provided as *Extended data* in English) ^27^. Part 1 is about demographic data. We collected data on gender, age, marital status, education, occupation, living duration in the community, distances between home and farmland, exposure to air pollution (i.e., cooking smoke, working in a factory with air pollution; asbestos, diesel engine exhaust, silica, wood dust, painting and welding exposure) and cigarette smoking. In Part 2, information on the historical use of pesticides were collected. In this study, pesticides were categorized into five groups: insecticides (organochlorine, organophosphate, carbamate, and pyrethoid), herbicides, fungicides, rodenticides, and molluscicides. For each groups of pesticides, we collected data on the numbers of years and days using pesticides. The data of lifetime pesticide exposure days were then computed by multiplying the total years of exposure by the number of days per year. This study also collected data on the use of 35 individual pesticides commonly found in Thailand.

Pesticide exposure data were collected by the researcher and two village health volunteers. Prior to data collection, all interviewers were trained on how to interview and properly use the questionnaire.

### Statistical analysis

Collected data were analysed using IBM SPSS Statistics (version 25) and OpenEpi (version 3.5.1). P values <0.05 were considered statistically significant. Demographic data was analysed using descriptive statistics. The associations were determined between lung cancer and groups of pesticides (herbicides, insecticides, fungicides, and molluscicides), between lung cancer and 17 individual compounds. Both crude and adjusted ORs with 95% confidence intervals (CIs) were presented. Adjusted ORs were analysed using multiple logistic regressions controlled for gender (male, female), age (≤54, 55–64, 65–74, and ≥75), cigarette smoking (ever, never smoke), occupation (farmer, non-farmer), and exposure to air pollution i.e., cooking smoke, and working in factory with air pollution (yes, no). In addition to the fundamental confounding factors, variables with statistically difference between cases and controls were included in a regression model.

Cumulative exposure days on groups of pesticides were categorized into quartiles (Q1-Q4; Q1 being the lowest exposure and Q4 the highest). The lung cancer risk was then predicted, using quartile 1 as a reference. For each specific pesticide, exposure data was categorized only to “ever used” and “never used”, but not the cumulative exposure days because number of subjects who reported using each pesticide was too small.

### Ethical considerations

This study was approved by the Ethics Board of Naresuan University (project number 550/60). Written informed consent was obtained from each subject before the interviewing process.

## Results

### Demographic information

In this study, most of study participants were male with a mean age of around 65. Both cases and controls have similar gender, age, marital status, education, occupation, period of residence, distances, pollution exposure, and cigarette smoking. More detailed demographic data among case and control groups were in Table 1 and in *Underlying data* ^28^.

**Table 1.** General characteristic of case and control groups.

Characteristic	Case		Control		P value ^**^
	n	%	n	%	
Total (any) (N = 680) ^*^	233	100.0	447	100.0	
Gender					0.693
Male	135	57.9	266	59.5	
Female	98	42.1	181	40.5	
Age (years)					0.891
≤54	34	14.6	71	15.9	
55–64	72	30.9	128	28.6	
65–74	72	30.9	146	32.7	
≥75	55	23.6	102	22.8	
Mean age (years) ± SD	66.04 ± 10.63		65.37 ± 10.88		
Median age (min–max)	65.00 (37–98)		66.00 (31–92)		
Marital status					0.644
Single	17	7.3	27	6.0	
Married	188	80.7	357	79.9	
Divorced/Separated	28	12.0	63	14.1	
Education completed					0.295
Primary school (Grade 1–6)	217	93.1	402	89.9	
Secondary school (Grade 7–12)	13	5.6	40	8.9	
Undergraduate or higher	3	1.3	5	1.2	
Occupation					0.970
Farmer	131	56.3	252	56.3	
Non-farmer	102	43.7	195	43.7	
Period of residence (years)					0.913
<21	25	10.7	45	10.1	
21–30	32	13.7	66	14.7	
>30	176	75.6	336	75.2	
Distances (m)					0.814
<500	102	43.8	197	44.1	
500–1,000	32	13.7	54	12.1	
>1,000	99	42.5	196	43.8	
Pollution exposure †					0.636
Yes	116	49.8	214	47.9	
No	117	50.2	233	52.1	
Cigarette smoking					0.207
Ever smoked	89	38.2	149	33.3	
Never smoked	144	61.8	298	66.7	

^*^N was 233 for case and 447 for control unless otherwise indicated. ^**^χ ^2^ test for categorical data; t-test for continuous data with statistically significant (p<0.05). †Cooking smoke, working in factory with air pollution, etc.

### Lung cancer and pesticide exposure

After adjusting for confounding factors, lung cancer was positively associated with historical exposure of study participants to herbicides, insecticides and fungicides ( Table 2). The adjusted variables included in the analysis were gender (male, female), age (≤54, 55–64, 65–74, ≥75), cigarette smoking (ever smoke, never smoke), occupation (farmer, non-farmer), and exposure to air pollution, i.e., cooking smoke, and working in factories with air pollution (yes, no). Compared with people in the lowest quartile (Q1) of number of days using herbicides, those in Q2-Q4 days of using herbicides had an elevated risk of lung cancer with odds ratio (OR) between 6.32 (95% CI 2.57–15.53) for people with Q2 exposure, and 22.18 (95% CI 9.14–53.80) for Q4 exposure (p < 0.001). A similar association was also found for days of insecticide use and lung cancer (OR = 2.79 for Q2, and OR = 28.43 for Q4, p < 0.001). For fungicides, only the Q4 group had a significant risk (OR = 4.97; 95% CI 1.49–16.56). For individual compounds, lung cancer was statistically associated with a historical use dieldrin (OR = 2.76; 95% CI 1.42–5.36), chlorpyrifos (OR = 3.98; 95% CI 2.06–7.67), and carbofuran (OR = 2.58; 95% CI 1.48–4.51) ( Table 3).

**Table 2.** Associations between type of pesticides use and lung cancer.

Pesticides use	Case		Control		OR (95% CI)	Adjusted OR (95% CI) *
	n	%	n	%		
Total	233	100.0	447	100.0		
Pesticides (any) (N = 490)						
*Herbicides (N = 347)*						
Yes	129	54.4	218	48.8	0.77 (0.56-1.06)	1.39 (0.95–2.03)
No	104	44.6	229	51.2		
*Number of years using herbicides (N = 347)*						
>20	23	17.8	51	23.4	1.32 (0.75–2.33)	1.42 (0.79–2.56)
11–20	76	58.9	107	49.1	1.18 (0.67–2.09)	1.34 (0.74–2.41)
1–10	30	23.3	60	27.5	Reference	Reference
P-value ***					0.320	0.201
*Number of days using herbicides (N = 347)*						
Q4 (>960)	52	40.3	30	13.8	16.95 (7.46–3.48)	22.18 (9.14–53.80) **
Q3 (529–960)	43	33.3	48	22.0	8.76 (3.94–19.49)	13.04 (5.38–31.58) **
Q2 (160–528)	25	19.4	52	23.9	4.70 (2.04–10.84)	6.32 (2.57–15.53) **
Q1 (<160)	9	7.0	88	40.4	Reference	Reference
P-value ***					<0.001	<0.001
*Insecticides (N = 314)*						
Yes	125	53.6	189	42.3	0.63 (0.46–0.87)	1.77 (1.22–2.57) ***
No	108	46.4	258	57.7		
*Number of years using the insecticides (N = 314)*						
>20	38	30.4	43	22.7	3.25 (1.69–6.27)	3.55 (1.79–7.04) **
11–20	72	57.6	96	50.8	1.83 (0.89–3.75)	1.99 (0.95–4.17)
1–10	15	12.0	50	26.5	Reference	Reference
P-value ***					<0.001	<0.001
*Number of days using the insecticides (N = 314)*						
Q4 (>1,200)	52	41.6	19	10.1	24.29 (9.87–59.75)	28.43 (11.11–72.76) **
Q3 (561–1,200)	45	36.0	40	21.2	9.98 (4.29–23.27)	9.47 (3.96–22.64) **
Q2 (231–560)	20	16.0	60	31.7	3.01 (1.24–7.32)	2.79 (1.12–6.95) **
Q1 (<231)	8	6.4	70	37.0	Reference	Reference
P-value ***					<0.001	<0.001
*Fungicides (N = 116)*						
Yes	42	18.0	74	16.6	0.90 (0.59–1.37)	1.08 (0.70–1.68)
No	191	82.0	373	83.4		
*Number of years using the fungicides (N = 116)*						
>20	7	16.6	10	13.5	0.41 (0.16–1.05)	2.24 (0.83–6.03)
11–20	23	54.8	39	52.7	0.53 (0.21–1.32)	0.79 (0.27–2.32)
1–10	12	28.6	25	33.8	Reference	Reference
P for trend					0.760	0.125
*Number of days using the fungicides (N = 116)*						
Q4 (>530)	16	38.1	13	17.6	3.39 (1.14–10.08)	4.97 (1.49–16.56) **
Q3 (161–530)	9	21.4	17	23.0	1.45 (0.46–4.57)	1.53 (0.46–5.06)
Q2 (96–160)	9	21.4	22	29.7	1.12 (0.37–3.45)	1.22 (0.37–4.01)
Q1 (<96)	8	19.1	22	29.7	Reference	Reference
P-value ***					0.014	0.051

*Logistic regression adjusted for gender, age (≤54, 55–64, 65–74, and ≥75), cigarette smoking (ever smoke and never smoke), occupation (farmer and non–farmer), and pollution exposure (i.e., cooking smoke, and working in factory with air pollution).

**Statistically significant (p <0.05).

***P-values for linear trends were derived using a continuous variable with midpoint value of each category.

**Table 3.** Associations between individual pesticide use and lung cancer.

Pesticide	Case		Control		OR (95% CI)	Adjusted OR (95% CI) *
	n	%	n	%		
Total	233	100.0	447	100.0		
Herbicides						
*Glyphosate*						
Yes	105	45.1	176	39.4	1.26 (0.91–1.74)	1.04 (0.61–1.76)
No	128	54.9	271	60.6		
*Paraquat*						
Yes	89	38.2	150	33.6	1.22 (0.88–1.70)	1.38 (0.84–2.24)
No	144	61.8	297	66.4		
*2, 4-Dichlorophenoxy acetic acid*						
Yes	47	20.2	70	15.7	1.36 (0.90–2.04)	1.25 (0.76–2.03)
No	186	79.8	377	84.3		
*Butachlor*						
Yes	14	6.0	24	5.4	1.12 (0.57–2.22)	0.32 (0.10–1.06)
No	219	94.0	423	94.6		
*Propanil*						
Yes	11	4.7	21	4.7	1.00 (0.47–2.12)	1.23 (0.37–4.09)
No	222	95.3	426	95.3		
*Alachlor*						
Yes	14	6.0	28	6.3	0.95 (0.49–1.85)	1.04 (0.45–2.44)
No	219	94.0	419	93.7		
**Insecticides**						
***Organochlorine insecticides***						
*Endosulfan*						
Yes	35	15.0	44	9.8	1.61 (1.00–2.60)	1.34 (0.71–2.52)
No	198	85.0	403	90.2		
*Dieldrin*						
Yes	24	10.3	20	4.5	2.45 (1.32–4.53)	2.76 (1.42–5.36) **
No	209	89.7	427	95.5		
*DDT*						
Yes	13	5.6	37	8.3	0.65 (0.34–1.25)	0.66 (0.31–1.42)
No	220	94.4	410	91.7		
***Organophosphate insecticides***						
*Chlorpyrifos*						
Yes	40	17.2	30	6.7	2.88 (1.74–4.76)	3.98 (2.06–7.67) **
No	193	82.8	417	93.3		
*Folidol*						
Yes	40	17.2	64	14.3	1.24 (0.80–1.90)	0.85 (0.47–1.54)
No	193	82.8	383	85.7		
*Mevinphos*						
Yes	16	6.9	22	4.9	1.42 (0.73–2.76)	0.53 (0.10–2.63)
No	217	93.1	425	95.1		
***Carbamate insecticides***						
*Carbaryl/Savin*						
Yes	14	6.0	34	7.6	0.77 (0.40–1.47)	0.78 (0.35–1.72)
No	219	94.0	413	92.4		
*Carbofuran*						
Yes	43	18.5	42	9.4	2.18 (1.37–3.45)	2.58 (1.48–4.51) **
No	190	81.5	405	90.6		
***Pyrethoid insecticides***						
*Abamectin*						
Yes	44	18.9	90	20.1	0.92 (0.61–1.37)	0.70 (0.43–1.16)
No	189	81.1	357	79.9		
**Fungicides**						
*Armure/Propiconazole*						
Yes	29	12.4	51	11.4	1.10 (0.67–1.79)	0.88 (0.46–1.69)
No	204	87.6	396	88.6		
*Methyl aldehyde*						
Yes	15	6.4	28	6.3	1.02 (0.53–1.96)	0.91 (0.43–1.92)
No	218	93.6	419	93.7		

*Logistic regression adjusted for gender, age (≤ 54, 55–64, 65–74, and ≥ 75), cigarette smoking (ever smoke and never smoke), occupation (farmer and non-farmer), and exposure to air pollution (i.e., cooking smoke, and working in factory with air pollution).

**Statistically significant (p < 0.05).

## Discussion

The study results showed a positive association between lung cancer and the historical use of herbicides, insecticides, and fungicides ( Table 3). The associations were closer for herbicides and insecticides, than for fungicides. For herbicides, compared to a group using the pesticides for less than 160 days (Q1), those in higher categories of days using herbicides all showed an elevated risk of lung cancer, with the highest among those using for more than 960 days (Q4) (OR = 22.18, 95% CI 9.14–53.80). A similar risk also presented among insecticide users. Lung cancer risk was found to increase in all categories of higher days using insecticides (Q2-Q4) with OR between 2.79 (95% CI 1.12–6.95) and 28.43 (95% CI 11.11–72.76). The highest category of years using insecticides (Q4) also showed a positive association with lung cancer (OR = 3.55; 95% CI 1.79–7.04). For fungicides, a significant association was found only among fungicide users at Q4 (>530 days) (OR = 4.97; 95% CI 1.49–16.56).

These results were consistent with literature indicating the potential carcinogenicity of pesticides ^29^. In an experimental study, exposure to pesticides caused the production of reactive oxygen species (ROS), an oxygen-containing species containing an unpaired electron, such as superoxide, hydrogen peroxide, and hydroxyl radical, which are highly unstable and may cause DNA damage, protein damage, mutagenicity, necrosis, and apoptosis ^30^. Pesticides may also increase the risk of cancer via other mechanisms including genotoxicity, tumour promotion, epigenetic effects, hormonal action and immunotoxicity ^31^. In epidemiological study, evidence linked pesticide exposure to lung cancer are increasing, and the issue will be further discussed in the following section.

For individual pesticides, the study found lung cancer to be statistically associated with dieldrin (OR = 2.76; 95 % CI 1.42–5.36), chlorpyrifos (OR = 3.98; 95% CI 2.06–7.67), and carbofuran (OR = 2.58; 95% CI 1.48–4.51) ( Table 3). Dieldrin is an extremely persistent organic pollutant linked to many health problems, e.g., Parkinson's disease, breast cancer, affecting the immunity system, the reproductive, and nervous systems ^32^. In the USA, the Agricultural Health Study found seven pesticides including dicamba, metolaclor, pendimethalin, carbofuran, chlorpyrifos, diazinon, and dieldrin to be positively associated with lung cancer ^14^. Further studies found dieldrin exposure to relate to the highest tertile of days use (RR = 5.30; 95% CI 1.50–18.60) ^33^. In Thailand, 688 tons of dieldrin was used in 1981–1990, before it was banned on May 16, 1990.

A study among pest control workers in Florida, USA found a long term exposure to organophosphate and carbamate insecticides to increase mortality risk of lung cancer (OR = 1.4; 95% CI 0.7–3.0) for subjects licensed from 10–19 year; OR = 2.1; 95% CI 0.8–5.5 for those licensed 20 year or more ^11^. In the Agricultural Health Study, a dose response relationships was found between lung cancer and chlorpyrifos (RR = 2.18; 95% CI 1.31–3.64) ^17^ and diazinon (RR = 3.46; 95% CI 1.57–7.65) ^18^. Similar results were also replicated in later studies of the Agricultural Health Study cohort for chlorpyrifos (RR = 1.80; 95% CI 1.00–3.23), which are referring to applicators in the lowest category of exposure ^17^. At this time, chlorpyrifos still not banned by Thai government. On the other hand, chlorpyrifos was the primary insecticide imported to Thailand (1,193,302 kilograms in 2013) ^34^.

For carbofuran, it has demonstrated mutagenic properties in laboratory studies ^3^. In the Agricultural Health Study, lung cancer risk of carbofuran for those with >109 days of lifetime exposure (RR = 3.05; 95% CI, 0.94–9.87) compared with those with < 109 lifetime exposure days ^35^. In Thailand, a study reported 87 different commercial brands of insecticides which were used for 202 rice fields in Suphanburi Province (abamectin 40%, followed by chlorpyrifos 30%, and carbofuran 20%), 93 brands of plant hormones, and 56 brands of chemicals for the control of plant diseases ^36^.

The major limitations of this case-control study were the recall bias where cases and controls can recall past exposure differently. Cases tend to memorize exposure better particularly when they know or are aware of exposure to cause their illness ^37^. However, with limited available information on the issue in Thailand, we did not expected participants to aware of pesticides as causal factor for lung cancer. In addition, data on pesticide exposure were obtained solely from the interview questionnaire without any exposure measurement. However, this information bias usually occurs evenly across a case and control group, and only has a negative effect on the association ^38^.

## Conclusion

This study found that the occurrence of lung cancer among people in Nakhon Sawan province, Thailand is associated with pesticide use. Out of 17 individual pesticides investigated, dieldrin, chlorpyrifos, and carbofuran showed significant associations with lung cancer incidence. These results are consistent with the literature from other parts of the world. Further studies should focus on identifying more individual pesticides that could cause lung cancer, as well as other types of cancer.

## Data availability

### Underlying data

Figshare: Pesticide and lung cancer. https://doi.org/10.6084/m9.figshare.12356270.v2 ^28^.

This project contains the following underlying data:

- Dataset_pesticide and lung cancer (SAV and CSV). (All underlying data gathered in this study.)
- Data Dictionary (DOCX).

### Extended data

Figshare: Questionnaire-pesticide and lung cancer Thailand. https://doi.org/10.6084/m9.figshare.12356384.v1 ^27^.

This project contains the following extended data:

- Questionnaire-pesticide and lung cancer Thailand (DOCX). (Study questionnaire in English.)

Data are available under the terms of the Creative Commons Zero “No right reserved” data waiver (CC0 1.0 Public domain dedication).
