# Supplementary material for: Pesticide exposure and lung cancer risk: A case-control study in Nakhon Sawan, Thailand
Source: F1000Res. 2024 Jan 4;9:492. Originally published 2020 Jun 2. [Version 8] doi: 10.12688/f1000research.24114.8 (PMC10904940; doi:10.12688/f1000research.24114.8)
Supplement: Peer review history

# Reviewer report: Reviewer response for version 8

**DOI:** 10.5256/f1000research.160692.r247032

**Published:** 2024-02-27

**Authors:** Adel S Girgis

**Affiliations:** 1 Department of Pesticide Chemistry, National Research Centre, Dokki, Giza, 12622, Egypt

The study describes the association of lung cancer with few numbers of pesticides through a questionnaire case controlled study.    

 The study is so useful for academic and applicable studies. However, little attention was mention for the mode of action associated with lung cancer due to exposure to pesticides. ROS was mentioned as the main element for this cancer type. However, other elements due to smoking and climatic pollution should also been taken into consideration, taking into account the increment of lung cancer is a worldwide problem. This may be considered in a future study.

Is the work clearly and accurately presented and does it cite the current literature?

Yes

If applicable, is the statistical analysis and its interpretation appropriate?

Yes

Are all the source data underlying the results available to ensure full reproducibility?

Yes

Is the study design appropriate and is the work technically sound?

Yes

Are the conclusions drawn adequately supported by the results?

Yes

Are sufficient details of methods and analysis provided to allow replication by others?

Yes

Reviewer Expertise:

Synthetic Organic Chemistry, Medicinal Chemistry, Computational Chemistry

I confirm that I have read this submission and believe that I have an appropriate level of expertise to confirm that it is of an acceptable scientific standard.

# Reviewer report: Reviewer response for version 8

**DOI:** 10.5256/f1000research.160692.r247037

**Published:** 2024-02-20

**Authors:** Md Sadique Hussain

**Affiliations:** 1 School of Pharmaceutical Sciences, Jaipur National University, Jaipur, India

**General Assessment:** The manuscript entitled "Pesticide exposure and lung cancer risk: A case-control study in Nakhon Sawan, Thailand" presents a well-written and well-designed study investigating the association between pesticide exposure and lung cancer risk in a specific geographic context. The study is methodologically sound, with clear objectives, detailed methods, robust statistical analysis, and meaningful results. The findings contribute to advancing the understanding of the health risks associated with pesticide use, particularly in regions where such investigations are limited. The manuscript is structured logically, with a comprehensive introduction, clear methods, and a concise presentation of results and conclusions. Overall, the manuscript provides valuable insights into the potential risks of pesticide exposure and lung cancer, and it is suitable for indexing pending minor revisions.

**Specific Comments:**

1. **Title and Abstract:** The title effectively summarizes the main topic and location of the study. The abstract provides a concise summary of the study, including background, methods, results, and conclusions. It effectively highlights the significance of the study's findings in the context of contributing to the literature on pesticide exposure and lung cancer.
2. **Introduction:** The introduction provides relevant background information on the prevalence and mortality rates of lung cancer globally and in Thailand. However, it would be beneficial to update the data on lung cancer prevalence and mortality rates to ensure the relevance and accuracy of the information provided (Hussain *et al.*, 2023 ^1^; Hussain *et al.*, 2024 ^2^).
3. **Methods:** The methods are clear and replicable, providing detailed descriptions of the study design, participant selection criteria, data collection process, questionnaire used, statistical analysis methods, and ethical considerations. The inclusion and exclusion criteria for cases are clearly described, and the participation rate is provided. The methods for calculating cumulative pesticide exposure days and categorizing exposure levels are explained thoroughly. Ethical considerations, including approval from the Ethics Board and obtaining written informed consent from participants, are appropriately addressed.
4. **Results:** The results are well-presented and consistent with the methods described. The associations between historical pesticide exposure and lung cancer risk, adjusted for various confounding factors, are statistically significant. The identification of specific individual compounds associated with an elevated risk of lung cancer adds to the novelty of the study's findings. The results align with previous research findings and contribute to advancing the understanding of pesticide exposure and lung cancer risk in the study's geographic context.
5. **Discussion:** The discussion effectively interprets the study findings in the context of existing literature and provides insights into the potential mechanisms underlying the observed associations between pesticide exposure and lung cancer risk. It discusses the implications of the study findings for future research directions and public health interventions.
6. **Conclusion:** The conclusion provides a clear summary of the main findings of the study and emphasizes the significance of addressing the potential health risks associated with pesticide exposure. It effectively highlights the need for further research to confirm the results and identify additional pesticides that could cause lung cancer.

**Overall Recommendation:** The manuscript is well-written and makes a valuable contribution to the field. I recommend accepting the manuscript for indexing pending minor revisions, including updating the introduction with the latest data on lung cancer prevalence and mortality rates.

Is the work clearly and accurately presented and does it cite the current literature?

Partly

If applicable, is the statistical analysis and its interpretation appropriate?

Yes

Are all the source data underlying the results available to ensure full reproducibility?

Yes

Is the study design appropriate and is the work technically sound?

Yes

Are the conclusions drawn adequately supported by the results?

Yes

Are sufficient details of methods and analysis provided to allow replication by others?

Yes

Reviewer Expertise:

Oncology, Lung cancer, Pharmacology, Non-coding RNAs, Cardiovascular Pharmacology, Toxicology

I confirm that I have read this submission and believe that I have an appropriate level of expertise to confirm that it is of an acceptable scientific standard.

## References

[1] : Long non-coding RNAs in lung cancer: Unraveling the molecular modulators of MAPK signaling. *Pathol Res Pract* .2023;249: 10.1016/j.prp.2023.154738 154738 10.1016/j.prp.2023.154738 37595448

[2] : Unlocking the secrets: Volatile Organic Compounds (VOCs) and their devastating effects on lung cancer. *Pathol Res Pract* .2024;255: 10.1016/j.prp.2024.155157 155157 10.1016/j.prp.2024.155157 38320440

# Reviewer report: Reviewer response for version 7

**DOI:** 10.5256/f1000research.148634.r226247

**Published:** 2023-12-23

**Authors:** Neela Guha

**Affiliations:** 1 California Environmental Protection Agency, Sacramento, California, USA

The authors report the results of a case-control study of lung cancer and pesticide exposure in Thailand.  This paper makes an important contribution to the literature, as most peer-reviewed publications on the topic come from developed Western countries. The authors should highlight this in the abstract and manuscript text. The reporting and structuring of the manuscript could be strengthened by following STROBE guidelines.

 The reporting on specific pesticides is helpful for risk assessment and to initiate public health actions to limit exposure to pesticides.  I have been asked to review starting on version 7 of the paper, which appears to have already been peer-reviewed. Although this paper has the potential to make an important contribution to the literature, there are a few concerns.

 Please further describe how controls were selected. How were neighbor controls identified and contacted? What was the percent participation? Were there efforts to assess the potential for selection bias? Savitz 2016 state: “Tools for evaluating the potential for selection bias in case-control studies include comparing measured exposure prevalence among controls to an external population and determining whether the exposure among controls follows expected patterns, examining exposure-disease associations in relation to markers of susceptibility to bias, adjusting for markers of selection, and evaluating whether expected associations between exposure and disease can be confirmed.” https://academic.oup.com/book/8266/chapter-abstract/153866697?redirectedFrom=fulltext In Table 1, what is the variable ‘Distances (m)’ signify?

 Selection of covariates in the model and potential confounders should be further described. Risk factors for lung cancer can be identified through several authoritative sources, including the IARC Monographs and Cogliano et al (JNCI). The file on the list of classifications by cancer site can guide you to the individual monographs. https://monographs.iarc.who.int/agents-classified-by-the-iarc/ Justification for covariates and model selection could be provided with a directed acyclic graph (DAG) and a correlation matrix to examine correlation between the covariates (including pesticides).

 Statistical analysis: Why was conditional logistic regression used, given this was not a individually pair-matched design. For the categorical analyses in table 2, why are the risk estimates <1 in Q1?

Is the work clearly and accurately presented and does it cite the current literature?

Partly

If applicable, is the statistical analysis and its interpretation appropriate?

Partly

Are all the source data underlying the results available to ensure full reproducibility?

Yes

Is the study design appropriate and is the work technically sound?

Partly

Are the conclusions drawn adequately supported by the results?

Partly

Are sufficient details of methods and analysis provided to allow replication by others?

Yes

Reviewer Expertise:

cancer epidemiology, occupational and environmental epidemiology, meta-analysis, bias analysis

I confirm that I have read this submission and believe that I have an appropriate level of expertise to confirm that it is of an acceptable scientific standard, however I have significant reservations, as outlined above.

# Reviewer report: Reviewer response for version 6

**DOI:** 10.5256/f1000research.140396.r154761

**Published:** 2022-11-02

**Authors:** Ann C Olsson

**Affiliations:** 1 Environment and Lifestyle Epidemiology Branch, International Agency for Research on Cancer, Lyon, France

I have read the new version and I still have reservations for approving this paper for publication.

*"The minimum sample size was determined to be 229 for cases and 458 for controls using Kelsey’s formula21 (unmatched population base case-control study). The assumptions used were as follows: proportion of case with pesticide exposure was 0.522, proportion of control with exposure was 0.423, and the ratio of case to control was 1:224."*

 Comment: The minimum sample size for what? This paragraph is missing something important such as “The minimum sample size to detect an OR of 1.5 was determined to be ….”

 The questionnaire on-line does not seem to be the one used for data collection because cooking fume and occupational air pollution is one question in the questionnaire and is reported separately in the table. The question regarding smoking in the questionnaire does not allow to calculate cumulative consumption. It seems that the authors “updated” the questionnaire following my previous comments and this is not correct. The questionnaire should be the one actually used for the data collection.

 In “Demographic information” Cases and controls **do not** have similar cigarette smoking.

 The authors completely ignore co-exposures, i.e., are those exposed to ”x” also exposed to “y” and “z”? If so, it is not possible to conclude that one agent is associated with lung cancer. There are several ways to address this, but difficult in a relatively small case-control study like this one.

Is the work clearly and accurately presented and does it cite the current literature?

Partly

If applicable, is the statistical analysis and its interpretation appropriate?

Not applicable

Are all the source data underlying the results available to ensure full reproducibility?

Yes

Is the study design appropriate and is the work technically sound?

Partly

Are the conclusions drawn adequately supported by the results?

Partly

Are sufficient details of methods and analysis provided to allow replication by others?

Partly

Reviewer Expertise:

NA

I confirm that I have read this submission and believe that I have an appropriate level of expertise to confirm that it is of an acceptable scientific standard, however I have significant reservations, as outlined above.

# Response

**Published:** 2023-05-23

**Authors:** Chudchawal Juntarawijit

**Affiliations:** Naresuan University, Thailand

**Response to reviewer**

**Reviewer's note:** *"I have read the new version and I still have reservations for approving this paper for publication."*

*"The minimum sample size was determined to be 229 for cases and 458 for controls using Kelsey’s formula21 (unmatched population base case-control study). The assumptions used were as follows: proportion of case with pesticide exposure was 0.522, proportion of control with exposure was 0.423, and the ratio of case to control was 1:224.*

*Comment: The minimum sample size for what? This paragraph is missing something important such as “The minimum sample size to detect an OR of 1.5 was determined to be ….”"*

**Response**: **First, we would like to thank you very much for your constructive comments and suggestions.**

**The statement has been revised as suggested. The new statement will be ... "The minimum sample size to detect an OR of 1.6 was determined to be 215 for cases and 430 for controls based on Kelsey’s formula..."**

**Comment**: *"The questionnaire on-line does not seem to be the one used for data collection because cooking fume and occupational air pollution is one question in the questionnaire and is reported separately in the table. The question regarding smoking in the questionnaire does not allow to calculate cumulative consumption. It seems that the authors “updated” the questionnaire following my previous comments and this is not correct. The questionnaire should be the one actually used for the data collection."*

**Response**: **In the actual used questionnaire, information on exposure to cooking fumes and air pollution was collected using two separate questions. However, in the first version, the data were combined as a single variable when running the regression model so the questionnaire was changed according to the data.  The problem occurs because we decided to use the original data as suggested by reviewers but not an updated the questionnaire. Concerning cigarette smoking, we actually had the data on number and duration to calculate cumulative consumption, but, in the first draft, the information was not used and thus was not included in the questionnaire. Sorry for not updating the questionnaire.**

**Comment**: *"In “Demographic information” Cases and controls **do not** have similar cigarette smoking."*

**Response**: **Thank you for the notice. The error occurred because a new analysis using more detailed data on cigarette smoking yielded a higher smoking rate for cases, but we forgot to update the results.**

**The mistake has been corrected by adding the following statement:**

**"...However, the cases had about twice the proportion of those who reported ever smoking a cigarette (23.6%) compared to the controls (13.6%). ..."**

**Comment**: *"The authors completely ignore co-exposures, i.e., are those exposed to ”x” also exposed to “y” and “z”? If so, it is not possible to conclude that one agent is associated with lung cancer. There are several ways to address this, but difficult in a relatively small case-controlled study like this one."*

**Response**: **That is a great point. To find the potential effects of co-exposures, we decided to run a correlation matrix between those five individual pesticides with significant OR but found only paraquat and glyphosate to have a high correlation (correlation coefficients ≥0.30). However, these two pesticides did not have a significant OR with lung cancer, therefore the problem should not be a big concern.**

# Reviewer report: Reviewer response for version 4

**DOI:** 10.5256/f1000research.55470.r81706

**Published:** 2021-04-26

**Authors:** Matthew R Bonner

**Affiliations:** 1 Department of Epidemiology and Environmental Health, School of Public Health and Health Professions, University at Buffalo, Buffalo, NY, USA

The conclusion that the significant associations observed for chlorpyrifos and carbonfuran are consistent with the literature from other parts of the world seems to be inaccurate and inconsistent with the early discussion of these chemicals.

Is the work clearly and accurately presented and does it cite the current literature?

Partly

If applicable, is the statistical analysis and its interpretation appropriate?

No

Are all the source data underlying the results available to ensure full reproducibility?

Yes

Is the study design appropriate and is the work technically sound?

Partly

Are the conclusions drawn adequately supported by the results?

Partly

Are sufficient details of methods and analysis provided to allow replication by others?

No

Reviewer Expertise:

Cancer epidemiology, pesticides, air pollution, occupational and environmental epidemiology

I confirm that I have read this submission and believe that I have an appropriate level of expertise to confirm that it is of an acceptable scientific standard, however I have significant reservations, as outlined above.

# Response

**Published:** 2021-04-28

**Authors:** Chudchawal Juntarawijit

**Affiliations:** Naresuan University, Thailand

**Response to reviewer**

 Comment:

 The conclusion that the significant associations observed for chlorpyrifos and carbofuran are consistent with the literature from other parts of the world seems to be inaccurate and inconsistent with the early discussion of these chemicals.

 Response:

 First of all, we would like to thank you very much for your informative comments and suggestions. I and my Ph.D. student have learned a lot from you.

 Yes, we agree that the conclusion was inconsistent with the discussion. Therefore, it was amended as follows:

**Conclusion**

 This study found that the occurrence of lung cancer among people in Nakhon Sawan province, Thailand is associated with pesticide use. Out of 17 individual pesticides investigated, three insecticides (dieldrin, chlorpyrifos, and carbofuran), and two herbicides (glyphosate, and paraquat) were associated with incidences of lung cancer. These results were moderately supported by the literature. More studies are still required to confirm the results and to identify more individual pesticides that could cause lung cancer, as well as other types of cancer. These issues should receive more attention since these chemicals have been used widely.

# Response

**Published:** 2021-09-17

**Authors:** Chudchawal Juntarawijit

**Affiliations:** Naresuan University, Thailand

Comment:

 The conclusion that the significant associations observed for chlorpyrifos and carbonfuran are consistent with the literature from other parts of the world seems to be inaccurate and inconsistent with the early discussion of these chemicals.

 Response:

 Would you please clarify the point and suggest which statement should be revised and how to properly state it.

# Reviewer report: Reviewer response for version 3

**DOI:** 10.5256/f1000research.54635.r79943

**Published:** 2021-03-12

**Authors:** Matthew R Bonner

**Affiliations:** 1 Department of Epidemiology and Environmental Health, School of Public Health and Health Professions, University at Buffalo, Buffalo, NY, USA

Much of the discussion still relies on older reports from the AHS. Even though reference 35 was added as a citation to the sentence on carbofuran, this AHS report details results for a considerable number of pesticides that overlap with pesticides reported on in this manuscript.

Is the work clearly and accurately presented and does it cite the current literature?

Partly

If applicable, is the statistical analysis and its interpretation appropriate?

No

Are all the source data underlying the results available to ensure full reproducibility?

Yes

Is the study design appropriate and is the work technically sound?

Partly

Are the conclusions drawn adequately supported by the results?

Partly

Are sufficient details of methods and analysis provided to allow replication by others?

No

Reviewer Expertise:

Cancer epidemiology, pesticides, air pollution, occupational and environmental epidemiology

I confirm that I have read this submission and believe that I have an appropriate level of expertise to confirm that it is of an acceptable scientific standard, however I have significant reservations, as outlined above.

# Response

**Published:** 2021-03-14

**Authors:** Chudchawal Juntarawijit

**Affiliations:** Naresuan University, Thailand

Comment:

 Much of the discussion still relies on older reports from the AHS. Even though reference 35 was added as a citation to the sentence on carbofuran, this AHS report details results for a considerable number of pesticides that overlap with pesticides reported on in this manuscript.

 Response:

 Thank for the suggestion. More information from the latest AHS study (reference 35) has been added to the discussion section.

# Reviewer report: Reviewer response for version 2

**DOI:** 10.5256/f1000research.31201.r76351

**Published:** 2021-01-18

**Authors:** Matthew R Bonner

**Affiliations:** 1 Department of Epidemiology and Environmental Health, School of Public Health and Health Professions, University at Buffalo, Buffalo, NY, USA

Overall, the revised manuscript is responsive to many of my comments. However, there are several outstanding issues that require additional attention.

**1) Response:** Matching of a few variables can be considered loose-matching, therefore, it is more appropriate to analyze using unconditional logistic regression. Kuo and team (2018) said that “There is a presumption that matched data need to be analyzed by matched methods. Conditional logistic regression has become a standard for matched case–control data to tackle the sparse data problem. The sparse data problem, however, may not be a concern for loose-matching data when the matching between cases and controls are not unique, and one case can be matched to other controls without substantially changing the association. Data matched on a few demographic variables are clearly loose-matching data, and we hypothesize that unconditional logistic regression is a proper method to perform.” (Kuo, Duan & Grady, 2018)*

 The characterization of matching on weak confounding factors as “loose-matching” seems to overlook the bias introduced by matching on confounders. As Neil Pearce states:

 “In essence, the matching process makes the controls more similar to the cases not only for the matching factor but also for the exposure itself. This introduces a bias that needs to be controlled in the analysis” (Pearce N. Analysis of matched case-control studies. *BMJ* (2016)). ^1^

 This is a well-recognized consequence of matching in case-control studies and mitigating this introduced bias in the analysis is fundamental to conducting a matched case-control study. The sparse data problem that is emphasized in the response is a common problem for most studies regardless of whether matching procedures were used. The use of conditional logistic regression can help with both these issues and should not be ignored because of a presumption of “loose-matching.” A comparison between the proportional hazards risk estimates with the unconditional logistic regression risk estimates seems to indicate that little if any bias was introduced by matching. This is likely due to a weak association between the matching factors and exposure. The methods section should state that conditional and unconditional logistic regression were used, that the results were similar, and results from the unconditional logistics regression are reported.   

**2)** **Response**: *In this study, we actually collected data from 35 individual pesticides, but 17 of them were excluded due to small sample size (less than 5 in each cell). Therefore, the OR groups may be larger than the individual OR ones.*

 This issue is unresolved, and depending on which table or exposure metric, one can render different conclusions about the association between pesticides and lung cancer. In table 2, herbicide days of use has a strong monotonic exposure response gradient, but in table 3, none of the reported herbicides have an association with lung cancer.

**Additional Comment:**

 1) The discussion section on chlorpyrifos and carbofuran describes results from the Agricultural Health Study. However, cited reports are not the most recent reports on these pesticides and lung cancer. A more recent report from 2017, found no association with either chlorpyrifos or carbofuran. The discussion should be updated in light of this more recent report ( https://ehp.niehs.nih.gov/doi/10.1289/EHP456). ^2^

Is the work clearly and accurately presented and does it cite the current literature?

Partly

If applicable, is the statistical analysis and its interpretation appropriate?

No

Are all the source data underlying the results available to ensure full reproducibility?

Yes

Is the study design appropriate and is the work technically sound?

Partly

Are the conclusions drawn adequately supported by the results?

Partly

Are sufficient details of methods and analysis provided to allow replication by others?

No

Reviewer Expertise:

Cancer epidemiology, pesticides, air pollution, occupational and environmental epidemiology

I confirm that I have read this submission and believe that I have an appropriate level of expertise to state that I do not consider it to be of an acceptable scientific standard, for reasons outlined above.

## References

[1] : Authors' response: Mezei et al's "Comments on a recent case-control study of malignant mesothelioma of the pericardium and the tunica vaginalis testis". *Scand J Work Environ Health* .2021;47(1) : 10.5271/sjweh.3910 87-89 10.5271/sjweh.3910 33388783 PMC7801133

[2] : Occupational Exposure to Pesticides and the Incidence of Lung Cancer in the Agricultural Health Study. *Environ Health Perspect* .125(4) : 10.1289/EHP456 544-551 10.1289/EHP456 27384818 PMC5381995

# Response

**Published:** 2021-02-10

**Authors:** Chudchawal Juntarawijit

**Affiliations:** Naresuan University, Thailand

**Comment:**

 Overall, the revised manuscript is responsive to many of my comments. However, there are several outstanding issues that require additional attention.

**1) Response:** Matching of a few variables can be considered loose-matching, therefore, it is more appropriate to analyze using unconditional logistic regression. Kuo and team (2018) said that “There is a presumption that matched data need to be analyzed by matched methods. Conditional logistic regression has become a standard for matched case–control data to tackle the sparse data problem. The sparse data problem, however, may not be a concern for loose-matching data when the matching between cases and controls are not unique, and one case can be matched to other controls without substantially changing the association. Data matched on a few demographic variables are clearly loose-matching data, and we hypothesize that unconditional logistic regression is a proper method to perform.” (Kuo, Duan & Grady, 2018)*

 The characterization of matching on weak confounding factors as “loose-matching” seems to overlook the bias introduced by matching on confounders. As Neil Pearce states:

 “In essence, the matching process makes the controls more similar to the cases not only for the matching factor but also for the exposure itself. This introduces a bias that needs to be controlled in the analysis” (Pearce N. Analysis of matched case-control studies. *BMJ* (2016)). ^1^

 This is a well-recognized consequence of matching in case-control studies and mitigating this introduced bias in the analysis is fundamental to conducting a matched case-control study. The sparse data problem that is emphasized in the response is a common problem for most studies regardless of whether matching procedures were used. The use of conditional logistic regression can help with both these issues and should not be ignored because of a presumption of “loose-matching.” A comparison between the proportional hazards risk estimates with the unconditional logistic regression risk estimates seems to indicate that little if any bias was introduced by matching. This is likely due to a weak association between the matching factors and exposure. The methods section should state that conditional and unconditional logistic regression were used, that the results were similar, and results from the unconditional logistics regression are reported.   

**Response:**

 The statement that both conditional and unconditional analyses were performed has been added to the method section.

**Comment:**

**2) Response**: *In this study, we actually collected data from 35 individual pesticides, but 17 of them were excluded due to small sample size (less than 5 in each cell). Therefore, the OR groups may be larger than the individual OR ones.*

 This issue is unresolved, and depending on which table or exposure metric, one can render different conclusions about the association between pesticides and lung cancer. In table 2, herbicide days of use has a strong monotonic exposure response gradient, but in table 3, none of the reported herbicides have an association with lung cancer.

**Response:**

 Taking the suggestion, we decided to recheck the data and found some individual pesticides to have enough sufficient participants to calculate cumulative exposure days. Reanalysis of the data using quartile of exposure days, found good results. Those exposed to glyphosate and paraquat in Q4 and Q3 had a significant association with lung cancer. So, the issue was solved. Thank you very much for your thoughtful suggestions. Without it, we would miss this important finding.

 Also, to make it more consistent, we decided to use ‘nonexposed’ as a reference instead of Q1, and the reanalysis of the data. This change caused only a minor change in the results.

 All parts of the paper (abstract, results, and discussion section) were updated.

**Comment:**

**Additional Comment:**

 1) The discussion section on chlorpyrifos and carbofuran describes results from the Agricultural Health Study. However, cited reports are not the most recent reports on these pesticides and lung cancer. A more recent report from 2017, found no association with either chlorpyrifos or carbofuran. The discussion should be updated in light of this more recent report ( https://ehp.niehs.nih.gov/doi/10.1289/EHP456). ^2^

**References**

 1. Marinaccio A, Consonni D, Mensi C, Mirabelli D, et al.: Authors' response: Mezei et al's "Comments on a recent case-control study of malignant mesothelioma of the pericardium and the tunica vaginalis testis". *Scand J Work Environ Health*. 2021; **47** (1): 87-89 PubMed Abstract | Publisher Full Text

 2. Bonner MR, Freeman LE, Hoppin JA, Koutros S, et al.: Occupational Exposure to Pesticides and the Incidence of Lung Cancer in the Agricultural Health Study. *Environ Health Perspect*. **125** (4): 544-551 PubMed Abstract | Publisher Full Text

**Response:**

 The reference has been updated. Thank you for the information.

# Reviewer report: Reviewer response for version 1

**DOI:** 10.5256/f1000research.26600.r73264

**Published:** 2020-10-30

**Authors:** Matthew R Bonner

**Affiliations:** 1 Department of Epidemiology and Environmental Health, School of Public Health and Health Professions, University at Buffalo, Buffalo, NY, USA

The manuscript reports the results of a case-control study designed to investigate exposure to pesticides and lung cancer. Cases and controls were recruited between January 1, 2014, and March 31, 2017, from Nakhon Sawan Province, Thailand. Controls were matched to cases on age and sex. Pesticide exposure was assessed with interviews with a structured questionnaire inquiring about days and years of pesticide use. Logistic regression, adjusting for potential confounders, was used to estimate the odds ratio and 95% confidence intervals. Pesticide classes (herbicides and organophosphate) and select specific pesticides were positively associated with lung cancer in this study. The authors conclude that “…lung cancer among Thai people in Nakhon Sawan province is associated with previous pesticide use.” Overall, the study seems to be designed well, but crucial information regarding several specific details are missing from the report. These details, and other concerns, are described below.

**Comments:**

1. Crucial information about the lung cancer cases is missing. Specifically, were the cases comprised of 1 ^st^ primary lung cancer or were lung cancer cases with a prior history of another cancer, including lung, eligible to participate in the study. Were the lung cancer cases’ diagnosis histologically confirmed? What was the stage and grade of these lung cancer cases? On average, how long after their diagnosis were lung cancer cases interviewed? 
2. Were potential controls excluded if they had a prior history of cancer?
3. The methods state that two neighbor controls were selected randomly. This implies that there a sampling frame of some sort. That sampling frame for random selection needs to be adequately described.
4. The controls were matched on age and sex to the cases. This necessitates a conditional logistic regression to account for the selection bias introduced by matching. Unconditional logistic regression is inappropriate for a matched case-control study. Breaking the matching and adjusting for the matching factors may not bias the odds ratios, but this should be confirmed by comparing ORs estimated with unconditional logistic regression and conditional logistic regression.
5. In table 2, there is striking qualitative confounding for the pesticide classes (yes vs. no). For instance, the crude OR organophosphates is 0.63 (95% CI = 0.46-0.87) while the adjusted OR is 1.77 (95% CI = 1.22-2.57). The use of unconditional logistic regression might explain this as the crude estimate for such a regression is inappropriate. That notwithstanding, a number of other variables were included in the regressions. Given this qualitative confounding, additional analyses to identify variables or combination of variables is driving this confounding is warranted.
6. Tobacco smoking is a recognized strong risk factor for lung cancer and a known potential confounder in studies of other exposures and lung cancer. As such, substantial efforts to mitigate confounder are often employed. In this study, smoking was a binary (ever vs. never) variable that may not adequately capture the interrelationship between pesticide use and lung cancer to control confounding. More detailed smoking information, if available, should be explored to determine the potential for residual confounding of the reported associations
7. In table 1, smoking is not associated with lung cancer. This suggests that selection forces in the recruitment of cases and controls are biasing the study results. It seems unusual that 61% of lung cancer cases were never smokers. Is this a typical feature of lung cancer in Thailand?
8. The results reported in tables 2 and 3 seem to be internally inconsistent. For instance, the ORs for organophosphates depicted in table 4 indicate a strong association with lung cancer with days of use (Q4 vs. Q1 OR = 28.43 (95% CI = 11.11-72.76); an extremely large magnitude. However, the ORs for specific organophosphate insecticides are much more modest, although the statistically significant associations with chlorpyfos and dielrin. A similar pattern is evident for herbicide as well. This lack of internal consistency really points to the methodological limitations as a likely explanation for the observed association.
9. Recall bias was discussed as a limitation, but nothing is mentioned about other threats to internal validity. For instance, the potential for selection bias to arise from the recruitment strategies. As mentioned above, the lack of an association with smoking seems to indicate something is awry. In addition, exposure misclassification is undoubtedly present and should be discussed in the Discussion along with the other potential limitations.
10. References 20 and 35 are the same report.

Is the work clearly and accurately presented and does it cite the current literature?

Partly

If applicable, is the statistical analysis and its interpretation appropriate?

No

Are all the source data underlying the results available to ensure full reproducibility?

Yes

Is the study design appropriate and is the work technically sound?

Partly

Are the conclusions drawn adequately supported by the results?

Partly

Are sufficient details of methods and analysis provided to allow replication by others?

No

Reviewer Expertise:

Cancer epidemiology, pesticides, air pollution, occupational and environmental epidemiology

I confirm that I have read this submission and believe that I have an appropriate level of expertise to state that I do not consider it to be of an acceptable scientific standard, for reasons outlined above.

# Response

**Published:** 2020-12-02

**Authors:** Chudchawal Juntarawijit

**Affiliations:** Naresuan University, Thailand

**Comment**:

 1. Crucial information about the lung cancer cases is missing. Specifically, were the cases comprised of 1 ^st^ primary lung cancer or were lung cancer cases with a prior history of another cancer, including lung, eligible to participate in the study. Were the lung cancer cases’ diagnosis histologically confirmed? What was the stage and grade of these lung cancer cases? On average, how long after their diagnosis were lung cancer cases interviewed? 

**Response**:

 The cases comprised of 1 ^st^ primary lung cancer. The cases were confirmed by Computerized Tomography scan (CT scan), Magnetic Resonance Imaging (MRI), ultrasound of the whole abdomen, and chest radiography or Chest X-ray (CXR), and histology of primary and metastasis. More information was added to Table 1, and provided in the Table below.

 On average, the patients were interviewed approximately1 year after they had been diagnosed with lung cancer.

**Table**. More information on morphology and stage of the study cases.

**Morphology **of lung cancer cases:

 Adenocarcinoma                                   114 (48.9)

 Small cell carcinoma                               21 (9.0)

 squamous cell carcinoma                        17 (7.3)

 Large cell carcinoma                                 9  (3.9)

 Neoplasm/ malignant                               68   (29.2)

 Unspecified                                                4   (1.7)

**Stage**

 IA, IB                                                14 (6.0)

 IIA, IIB                                             38 (16.3)

 IIIA, IIIB                                           57 (24.5)

 IV (Distant metastasis)                          0 (0)

 Unknown/ unspecified                   124 (53.2)

**Comment**:

 2. Were potential controls excluded if they had a prior history of cancer?

**Response**:

 Yes, potential controls were excluded if they had a prior history of cancer.

**Comment**:

 3. The methods state that two neighbor controls were selected randomly. This implies that there a sampling frame of some sort. That sampling frame for random selection needs to be adequately described.

**Response**:

 The control was, in fact, selected using convenience sampling. The information in the manuscript has been revised.

**Comment**:

 4. The controls were matched on age and sex to the cases. This necessitates a conditional logistic regression to account for the selection bias introduced by matching. Unconditional logistic regression is inappropriate for a matched case-control study. Breaking the matching and adjusting for the matching factors may not bias the odds ratios, but this should be confirmed by comparing ORs estimated with unconditional logistic regression and conditional logistic regression.

**Response**:

 Matching of a few variables can be considered loose-matching, therefore, it is more appropriate to analyze using unconditional logistic regression. Kuo and team (2018) said that “There is a presumption that matched data need to be analyzed by matched methods. Conditional logistic regression has become a standard for matched case–control data to tackle the sparse data problem. The sparse data problem, however, may not be a concern for loose-matching data when the matching between cases and controls are not unique, and one case can be matched to other controls without substantially changing the association. Data matched on a few demographic variables are clearly loose-matching data, and we hypothesize that unconditional logistic regression is a proper method to perform.” (Kuo, Duan & Grady, 2018)*

           We gained interesting information by analyzing some of the data using Cox regression. The comparison between unconditional analysis and the Cox regression, yielded similar results. (see Table below). Comparing OR between Cox regression and logistic regression).

**Table.** Comparing OR between Cox regression and logistic regression.

****

**Logistic regression** **Cox regression**

 Endosulfan                                            OR (cr ude)       1.61 (1.00–2.60)                               2.01 (1.12-3.63)

                                                               OR (adjusted)    1.60 (0.97–2.63)                              1.81 (0.99-3.33)

 Dieldrin

                                                               OR (crude)          2.45 (1.32–4.53)                             3.28 (1.57-6.85)

                                                               OR (adjusted)      2.56 (1.36–4.81)                             3.62 (1.70-7.68)

 Chlorpyrifos

                                                                 OR (crude)          2.88 (1.74–4.76)                             3.50 (1.91-6.41)

                                                                 OR (adjusted)      3.29 (1.93–5.61)                             3.85 (2.05-7.22)

 Carbofuran

                                                                  OR (crude)            2.18 (1.37–3.45)                             2.36 (1.43-3.89)

                                                                  OR (adjusted)        2.10 (1.28–3.42)                             2.38 (1.41-4.01)

****

 *Kuo C-L, Duan Y and Grady J (2018) Unconditional or Conditional Logistic Regression Model for Age-Matched Case–Control Data? *Front. Public Health* 6:57. doi: 10.3389/fpubh.2018.00057 

**Comment**:

 5. In table 2, there is striking qualitative confounding for the pesticide classes (yes vs. no). For instance, the crude OR organophosphates is 0.63 (95% CI = 0.46-0.87) while the adjusted OR is 1.77 (95% CI = 1.22-2.57). The use of unconditional logistic regression might explain this as the crude estimate for such a regression is inappropriate. That notwithstanding, a number of other variables were included in the regressions. Given this qualitative confounding, additional analyses to identify variables or combination of variables is driving this confounding is warranted.

**Response**:

 After re-categorizing the smoking variable, and correcting a mistake on the variable coding, the new analysis yielded more consistent results with crude OR at 1.35 (95%CI 0.98-1.86) and adjusted OR at 1.40 (95%CI 0.97-2.02).

 6. Tobacco smoking is a recognized strong risk factor for lung cancer and a known potential confounder in studies of other exposures and lung cancer. As such, substantial efforts to mitigate confounder are often employed. In this study, smoking was a binary (ever vs. never) variable that may not adequately capture the interrelationship between pesticide use and lung cancer to control confounding. More detailed smoking information, if available, should be explored to determine the potential for residual confounding of the reported associations

 We actually collected data on the amount of cigarettes and smoking duration of the study participants, and then the information was used to compute number of cigarettes smoked by them in their life time. After grouping smoking status into “never smoked”, “smoked <109500 cigarettes”, and “smoked ≥109500 cigarettes”, a significant difference between case and control was found. The data was then used for the analysis of the odds ratio.

**Comment**:

 7. In table 1, smoking is not associated with lung cancer. This suggests that selection forces in the recruitment of cases and controls are biasing the study results. It seems unusual that 61% of lung cancer cases were never smokers. Is this a typical feature of lung cancer in Thailand?

**Response**:

 Yes, 61% of the cases never smoked is acceptable. It was reported that smoking prevalence of Thai males decreased from 60% to 39%, and from 5% to 2.1% in females between 1991 and 2014 [1]. While a survey in 2017 reported a smoking prevalence of 20.7% of the total adult population over 15 years old [2].

 It was interesting to note that in this study, 49.2% of the cases were adenocarcinoma lung cancer which has a limited relation to cigarette smoking, whereas squamous cell, and small cell lung carcinoma are highly related to smoking [3, 4].    

 [1] Jeon J, Sriplung H, Yeesoonsang S, Bilheem S, Rozek L, and et al. Temporal Trends and Geographic Patterns of Lung Cancer Incidence by Histology in Thailand, 1990 to 2014. Journal of Global Oncology 2018.

 [2] *Tobacco Control Research* and Knowledge Management Center ( *TRC*). Annual Report of Thailand Tobacco Survey, 2018. Mahidol University. www.trc.or.th. 2018.

 [3] Wu K, Wong E, and Chaudhry S. Lung Cancer; Classification of invasive lung cancer. Clin Chest Med 2002; Mar; 23(1):65-81.

 [4] Limsila T, Mitacek EJ, Caplan LS, and Brunnemann KD. Histology and Smoking History of Lung Cancer Cases and Implications for Prevention in Thailand. Preventive Medicine 1994; 23:249-252.

**Comment**:

 8. The results reported in tables 2 and 3 seem to be internally inconsistent. For instance, the ORs for organophosphates depicted in table 4 indicate a strong association with lung cancer with days of use (Q4 vs. Q1 OR = 28.43 (95% CI = 11.11-72.76); an extremely large magnitude. However, the ORs for specific organophosphate insecticides are much more modest, although the statistically significant associations with chlorpyfos and dielrin. A similar pattern is evident for herbicide as well. This lack of internal consistency really points to the methodological limitations as a likely explanation for the observed association.

**Response**:

 In this study, we actually collected data from 35 individual pesticides, but 17 of them were excluded due to small sample size (less than 5 in each cell). Therefore, the OR groups may be larger than the individual OR ones.

**Comment**:

 9. Recall bias was discussed as a limitation, but nothing is mentioned about other threats to internal validity. For instance, the potential for selection bias to arise from the recruitment strategies. As mentioned above, the lack of an association with smoking seems to indicate something is awry. In addition, exposure misclassification is undoubtedly present and should be discussed in the Discussion along with the other potential limitations.

**Response**:

 The problems of selection bias and exposure misclassification has been further discussed in the manuscript as suggested. The problem of lack of association with smoking has already been solved.

**Comment**:

 10. References 20 and 35 are the same report.

**Rsponse**:

 The error has been corrected.

# Reviewer report: Reviewer response for version 1

**DOI:** 10.5256/f1000research.26600.r65521

**Published:** 2020-06-25

**Authors:** Ann C Olsson

**Affiliations:** 1 Environment and Lifestyle Epidemiology Branch, International Agency for Research on Cancer, Lyon, France

Dear authors,

 I was pleased to review your paper that describes a case-control study in Thailand including 233 incident lung cancer cases and 458 controls focusing on exposures to pesticides. Please find enclosed my comments for your consideration. In the:

**Introduction**, first paragraph, I think you mean “ **Polycyclic aromatic **hydrocarbons”? I would not call a paper from 1999 “Recent studies…..” because it’s >20 years old.

**Methods**, first paragraph, it’s a case-control study, not a case-control **led** study; it would be good to include a few more details such as any time limit for having resided in the province?; from where did the TCB receive cases?; were the diagnosis confirmed by some diagnostic tool? Please clarify if the 299 were contacted and 229 accepted, or if only 229 were contacted and accepted. I would be surprised if the latter, and wonder why the other were not contacted. We also wish to know the “participation rate” among the control subjects. Neighbours are not a random sample it’s a convenience sample. If you mean that the interviewer randomly selected control subjects among all neighbours you need to explain how this was done, e.g. within a distance from the house or “snowball” technique. Why do you adjust for farming (yes/no)? Please explain your rational. It does not make sense to me.

**Questionnaire**, the English questionnaire does not indicate that the number of days of pesticide use is per year, so it seems strange that lifetime exposure is calculated by multiplying years with days. Please also clarify if “exposure” refers to “personally mix or apply pesticides” only, or if it also includes working in the fields? Provide more details regarding the data collection e.g. were the interviewers employed for the study full-time, or were they students?, were there any quality control measures implemented, e.g. double interviews of a proportion of subjects, were the interviewers interviewing both cases and controls?

**Results**, it is very strange that there is not difference between cases and controls regarding smoking, if you have an explanation for this please discuss it later.

**Discussion**, I don’t think that “the association were **closer** for herbicides and insecticides”, possibly “stronger” or “more pronounced”, and I prefer “more days” rather than “higher days”.

 Among the limitations I think there is more to information bias, e.g. it is commonly difficult to assess exposure to specific chemicals because people don’t know the names or don’t recognize exposure. I must admit that I get suspicious that there are no missing in the data and no category for “don’t know” in the questionnaire. I would add potential selection bias to the discussion; although we don’t really know the participation rate among controls or how neighbours were selected, they are generally not an ideal control population.

 Disclaimer:

 Where authors/reviewers are identified as personnel of the International Agency for Research on Cancer / World Health Organization, the authors/reviewers alone are responsible for the views expressed in this article and they do not necessarily represent the decisions, policy or views of the International Agency for Research on Cancer / World Health Organization

Is the work clearly and accurately presented and does it cite the current literature?

Partly

If applicable, is the statistical analysis and its interpretation appropriate?

Not applicable

Are all the source data underlying the results available to ensure full reproducibility?

Yes

Is the study design appropriate and is the work technically sound?

Partly

Are the conclusions drawn adequately supported by the results?

Partly

Are sufficient details of methods and analysis provided to allow replication by others?

Partly

Reviewer Expertise:

Occupational cancer epidemiology, lung cancer, case-control studies, cohort studies

I confirm that I have read this submission and believe that I have an appropriate level of expertise to confirm that it is of an acceptable scientific standard, however I have significant reservations, as outlined above.

# Response

**Published:** 2020-12-02

**Authors:** Chudchawal Juntarawijit

**Affiliations:** Naresuan University, Thailand

**Comment: Introduction**, first paragraph, I think you mean “ **Polycyclic aromatic **hydrocarbons”? I would not call a paper from 1999 “Recent studies…..” because it’s >20 years old.

**Response**: The term was changed to polycyclic aromatic hydrocarbons.

**Comment**: **Methods**, first paragraph, it’s a case-control study, not a case-control **led** study; it would be good to include a few more details such as any time limit for having resided in the province?; from where did the TCB receive cases?; were the diagnosis confirmed by some diagnostic tool? Please clarify if the 299 were contacted and 229 accepted, or if only 229 were contacted and accepted. I would be surprised if the latter, and wonder why the other were not contacted. We also wish to know the “participation rate” among the control subjects. Neighbours are not a random sample it’s a convenience sample. If you mean that the interviewer randomly selected control subjects among all neighbours you need to explain how this was done, e.g. within a distance from the house or “snowball” technique. Why do you adjust for farming (yes/no)? Please explain your rational. It does not make sense to me.

**Response**: The mistake was corrected and more information on residency, TCB, and diagnostic confirmation was added to the Methods. The information of the number of cases was clarified; and information on the participation rate was also provided.

 For the question, why did we adjust for farming? Actually, we tried to adjust for occupations since it is very likely that they will be exposed to pesticides differently. At first, there were several types of occupations, but due to the small number of participants in each category, the groups were limited to “farmer” and “none-farmer”.  These two groups tended to have different risks of exposure to environmental pesticides, due to the nature of their work and physical health.

**Comment**:

**Questionnaire**, the English questionnaire does not indicate that the number of days of pesticide use is per year, so it seems strange that lifetime exposure is calculated by multiplying years with days. Please also clarify if “exposure” refers to “personally mix or apply pesticides” only, or if it also includes working in the fields? Provide more details regarding the data collection e.g. were the interviewers employed for the study full-time, or were they students?, were there any quality control measures implemented, e.g. double interviews of a proportion of subjects, were the interviewers interviewing both cases and controls?

**Response**:

 More information was added and the mistakes were corrected. In this study, “exposure” refers to “personally mixed and/or applied pesticides” only, not working in the field. More information of interviewers was added to the methods. There were no other quality control measures implemented.

**Comment: Results**, it is very strange that there is not difference between cases and controls regarding smoking, if you have an explanation for this please discuss it later.

**Response**: Data on cigarette smoke was reanalyzed and the difference was observed using a new category.

**Comment**:

**Discussion**, I don’t think that “the association was **closer** for herbicides and insecticides”, possibly “stronger” or “more pronounced”, and I prefer “more days” rather than “higher days”.

**Response**: The term “closer” was changed to “stronger”

**Comment**: Among the limitations, I think there is more to information bias, e.g. it is commonly difficult to assess exposure to specific chemicals because people don’t know the names or don’t recognize exposure. I must admit that I get suspicious that there are no missing in the data and no category for “don’t know” in the questionnaire. I would add potential selection bias to the discussion; although we don’t really know the participation rate among controls or how neighbours were selected, they are generally not an ideal control population.

**Response**:

 Yes, we agree that it was likely that some of participants could not recall or know the name of the pesticides used. If this type of bias occurs it would be equal between both the case and control groups, and minimize the association between exposure to pesticides and lung cancer. More information regarding bias was added to the Discussion section, and more information about the control group was added to the Methods. Those who could not recall or “don’t know” the name of the pesticides were categorized as “not used”.
